# Talar Neck Osteotomy for Treating Neurologic Cavovarus Foot in an Adult: A Case Report

**DOI:** 10.7759/cureus.92648

**Published:** 2025-09-18

**Authors:** Dan Moriwaki, Tomoyuki Nakasa, Yasunari Ikuta, Satoru Sakurai, Nobuo Adachi

**Affiliations:** 1 Department of Orthopaedic Surgery, Graduate School of Biomedical and Health Sciences, Hiroshima University, Hiroshima, JPN; 2 Department of Artificial Joints and Biomaterials, Graduate School of Biomedical and Health Sciences, Hiroshima University, Hiroshima, JPN

**Keywords:** achilles tendon lengthening, cavovarus foot, equinus deformity, first metatarsal osteotomy, joint-preserving surgery, spina bifida, talar neck osteotomy, tibialis posterior tendon transfer

## Abstract

Talar neck osteotomy (TNO) has been reported as one of the surgical options for cavovarus foot caused by residual congenital clubfoot and neurologic disease in children, or talar neck fracture. However, TNO is rarely reported to correct the neurologic cavovarus foot in adults. We present the case of a 29-year-old male with spina bifida who exhibited a flexible cavovarus deformity of the foot, associated pain during ambulation, and plantar callosities. Preoperative weight-bearing foot radiographs showed cavovarus alignment, and three-dimensional computed tomography demonstrated increased varus/plantarflexion of the talar neck. We performed joint-preserving reconstruction comprising TNO, anterolateral transfer of the tibialis posterior tendon, dorsiflexion of the first‑metatarsal osteotomy, and Achilles tendon lengthening. At 24 months, radiographs and clinical examination confirmed a reduction of cavovarus deformity and plantar callosities. Bony union was achieved at all osteotomy sites without signs of talar avascular necrosis. The Japanese Society for Surgery of the Foot hindfoot score improved from 55 to 90, the visual analog scale for pain also improved from 7 to 0, and the‑Self-Administered Foot Evaluation Questionnaire scores improved in all domains. TNO combined with tendon transfer and first‑ray and gastrosoleus procedures can be a useful option within a comprehensive, deformity-specific strategy for adult neurologic cavovarus feet.

## Introduction

Cavovarus foot is characterized by increased arch height, hindfoot varus (inward tilting of the heel), midfoot plantarflexion (downward tilting of the midfoot), and forefoot pronation/adduction (inward turning of the forefoot) [1]. The most common etiologies are neuromuscular, traumatic, residual congenital clubfoot, or idiopathic causes [2]. Among these, neuromuscular disorders are the most frequent, and Charcot-Marie-Tooth disease is particularly common, with cavovarus deformities reported in approximately two-thirds of affected patients [2]. Imbalances among the tibialis anterior, peroneus longus and brevis, and tibialis posterior muscles contribute to plantarflexion of the first ray and worsening hindfoot varus [3]. Patients with cavovarus deformity often report pain, callosity formation under the metatarsal heads, foot fatigue, difficulty with footwear, and lateral ankle instability [2]. Although individualized treatment is necessary due to the variety of underlying diseases and severities, the common treatment goal is to achieve a plantigrade, painless foot that can tolerate standard footwear. Surgical intervention is considered in cases that fail to respond to conservative management, including stretching, casting, insoles, and customized shoes. Reported procedures include soft-tissue release [4,5], tendon transfer or lengthening [3,4,6], osteotomy (bone-cutting procedure) [3-6], and arthrodesis (joint fusion) [7], most often in combination and tailored to deformity components. Talar neck osteotomy (TNO) has been described to correct adduction and varus of the talar head-neck segment, primarily in pediatric patients with residual clubfoot or neuromuscular deformity [8,9] and in post-traumatic malunion after talar neck fracture [10-12]. Evidence for TNO in adults with neurologic cavovarus deformity is limited. We report a case of an adult neurologic flexible cavovarus foot who achieved good clinical outcomes by a combination of TNO, anterolateral transfer of the posterior tibialis tendon (TPT), dorsiflexion first metatarsal osteotomy (DFMO), and Achilles tendon lengthening (ATL). This report aims to describe the surgical management and favorable outcome of an adult patient with neurologic cavovarus foot, highlighting the role of TNO as a joint-preserving option.

## Case presentation

A 29-year-old male had progressive left-sided cavovarus deformity, pain during ambulation, and plantar callosities despite bracing. The patient reported a history of spina bifida, and he had a dysplasia of the left lower extremity since childhood. He had undergone deformity correction with an Ilizarov external fixator at age 9, followed by rehabilitation and orthotic therapy, with subsequent recurrence in adulthood.

**Figure 1:**
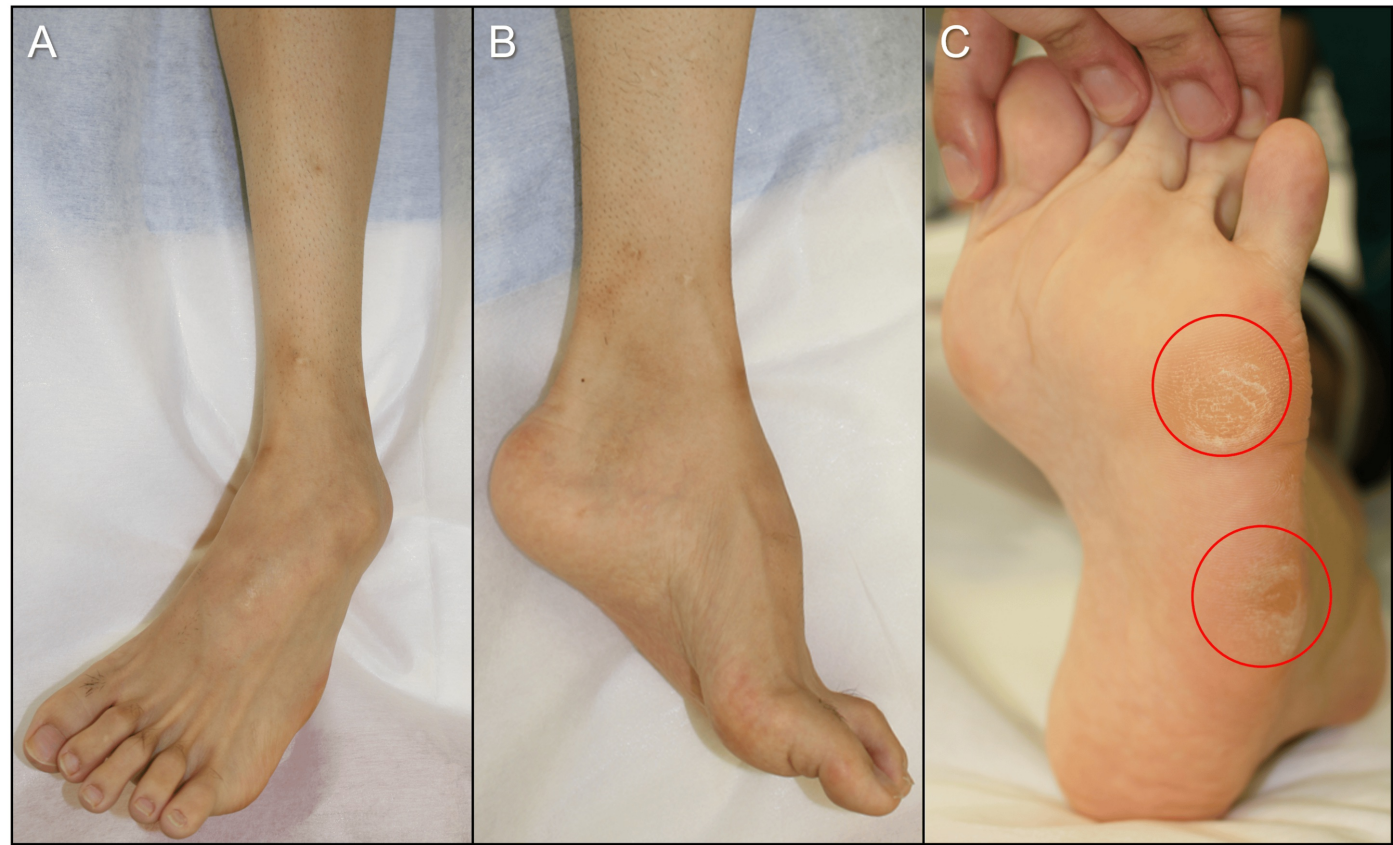
Preoperative photographs of the left foot and ankle. (A) Frontal ankle; (B) lateral foot; (C) plantar view (circle indicating callosity).

Physical examination revealed a left flexible cavovarus foot with painful callosities beneath the head and base of the fifth metatarsal (Figures 1A-1C).

**Figure 2:**
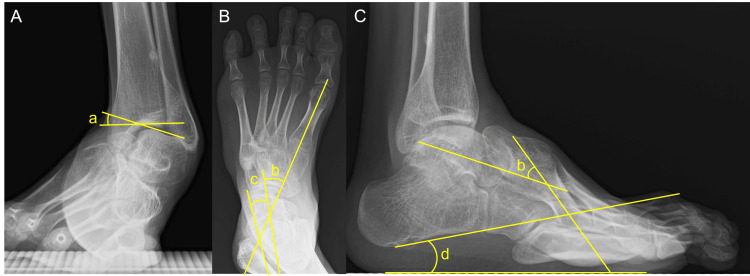
Preoperative weight-bearing radiographs (A) Anteroposterior view of the ankle; (B) anteroposterior view of the foot; (C) lateral view of the foot. (a) Talar tilt angle; (b) talo-first metatarsal angle; (c) talo-calcaneal angle; (d) calcaneal pitch angle

On manual muscle testing, the left foot and ankle exhibited preserved plantarflexion and eversion, but weakness in dorsiflexion and inversion. There were contractures of the Achilles tendon and tibialis posterior tendon (PTT). The range of motion (ROM) of the left ankle joint was -15° in dorsiflexion and 40° in plantarflexion with the knee extended. The Silfverskiöld test showed dorsiflexion of -10° with the knee flexed, indicating contracture of the gastro-soleus complex. Weight‑bearing radiographs revealed the cavovarus foot deformity, with a talar tilt angle of 20.7°(normal range; -1°-1°) on the anteroposterior (AP) view of the ankle, a talo-calcaneal angle of 7.2°(15°-27°) and a talo-first metatarsal angle of -31.7°(3°-11°) on the AP view of the foot, and a talo-first metatarsal angle of 37.1°(2°-10°) and a calcaneal pitch angle of 9.6°(13°-23°) on the lateral view of the foot (Figures 2A-2C) [13].

**Figure 3:**
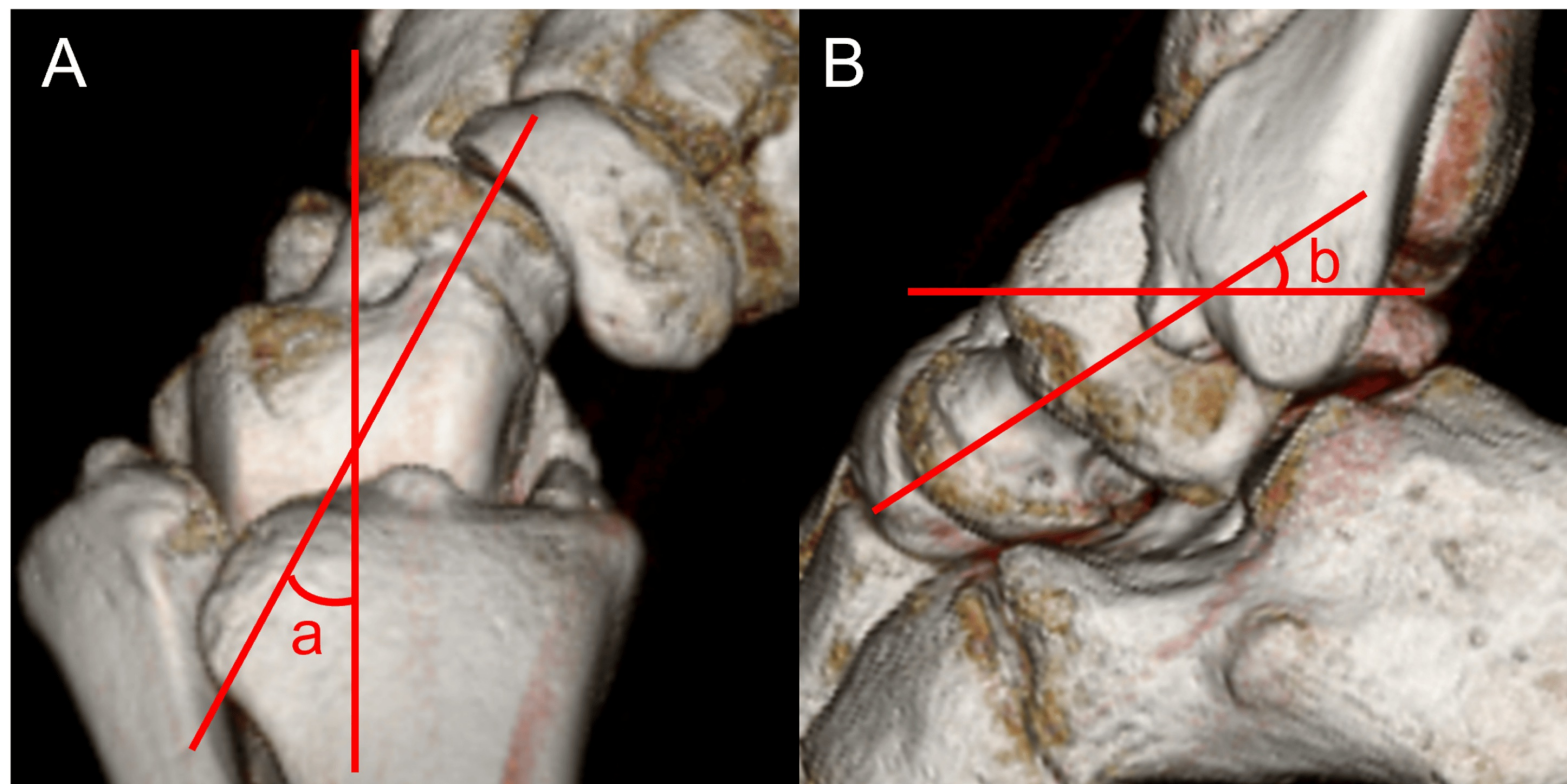
Three-dimensional computed tomography of the talus (A) Axial view, (B) sagittal view. (a) The declination angle of the talar neck relative to the body; (b) the inclination angle of the talar neck relative to the body

Three-dimensional computed tomography (3D-CT) demonstrated that the declination and inclination angles of the talar neck relative to the body were 32.9° and 29.3°, respectively, both greater than reported averages (Figures 3A-3B) [14].

The visual analog scale (VAS) was 7 points. The Japanese Society for Surgery of the Foot (JSSF) hindfoot scale was 55 points [15], and the Self-Administered Foot Evaluation Questionnaire (SAFE-Q) scores were 33.9 for pain, 52.3 for physical function, 25.0 for social function, 50.0 for shoe-related, and 30.0 for general health [16].

**Figure 4:**
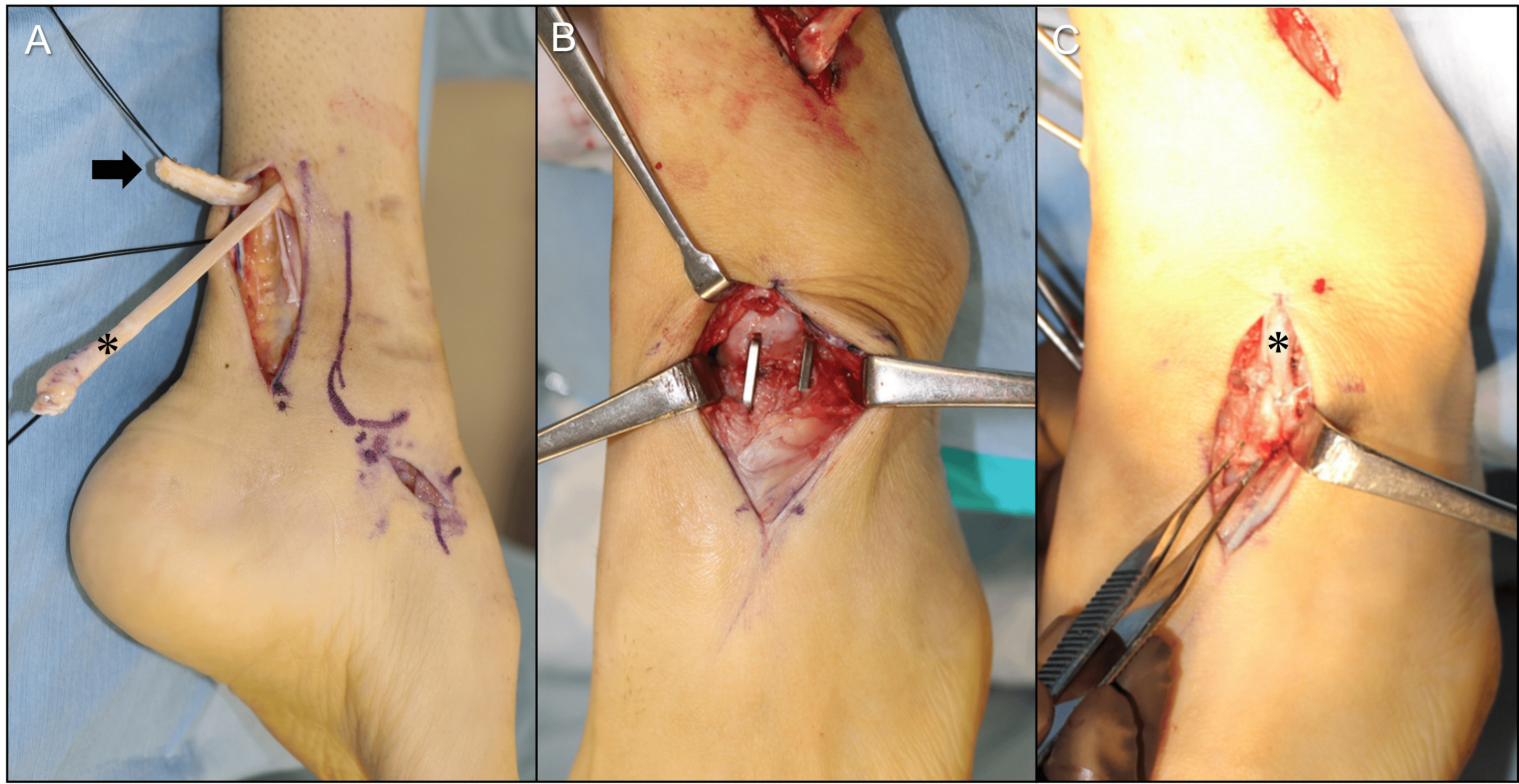
Intraoperative images (A) Proximal Achilles tendon (arrow) and tibialis posterior tendon (*) delivered retrograde; (B) talar neck after osteotomy; (C) tibialis posterior tendon (*) fixed to the lateral navicular.

We diagnosed his condition as a left neurologic flexible cavovarus foot caused by spina bifida. Differential diagnoses such as residual congenital clubfoot, post-traumatic deformity, and Charcot-Marie-Tooth disease were considered; however, these were excluded based on the patient’s history of spina bifida and the absence of trauma, congenital clubfoot, or familial neuropathy. The diagnosis of neurologic cavovarus foot was further supported by characteristic features, including a high medial arch, hindfoot varus, forefoot pronation/adduction, plantar callosities, and muscle imbalance on examination. Under general anesthesia, with the patient in the supine position and a thigh tourniquet applied, joint-preserving reconstruction was performed in the following sequence. First, Achilles tendon dissection was performed through a 6 cm skin incision on the posteromedial aspect of the ankle. The Achilles tendon was exposed and cut into a 6 cm Z-shaped section. Second, we performed the TPT procedure as described in a previous report [6]. A 3 cm longitudinal incision was made over the medial aspect of the navicular bone. The PTT was detached from its insertion, retrogradely pulled out through the ATL incision (Figure 4A).

The PTT was routed through a window in the interosseous membrane from medial to lateral and brought to the anterior compartment. Third, the TNO procedure was performed. A 5 cm longitudinal incision was made over the talonavicular joint. The talar neck was exposed with meticulous protection of its vascular supply. A closing‑wedge osteotomy (base lateral and dorsal) corrected varus and plantarflexion. The distal fragment was abducted and dorsiflexed, and fixation was achieved using two compression staples (DynaNite, 15 mm × 15 mm, Arthrex, Inc., Naples, USA) (Figure 4B). Fourth, the transferred PTT was passed under the retinaculum to the dorsum of the foot. Since PTT was not long enough to be fixed to the middle or lateral cuneiform, it was fixed into a lateral navicular tunnel using a 4.0-mm diameter interference screw with 10 mm length (BioComposite Tenodesis Screw, Arthrex, Inc., Naples, USA) (Figure 4C). Fifth, the Z-sectioned Achilles tendon was repaired with non-absorbable sutures with a 25 mm overlap, resulting in 35 mm lengthening. Sixth, DFMO was performed through a 3 cm longitudinal incision over the base of the first tarsometatarsal (TMT) joint. A closing-wedge osteotomy was made 15 mm distal to the first TMT joint, and fixation was achieved using a DynaNite (15 mm × 12 mm). Finally, after manual correction of the talar tilt, two 1.8-mm diameter Kirschner wires were inserted from the medial malleolus to the talar dome for temporary ankle fixation. Postoperative protocol included immobilization with a short-leg cast for 4 weeks, with non-weight-bearing precautions. At 4 weeks, Kirschner wires were removed, and a heeled short-leg cast allowed partial weight-bearing. At 6 weeks, the cast was discontinued with initiation of ROM exercise, strength training, and full weight-bearing.

**Figure 5:**
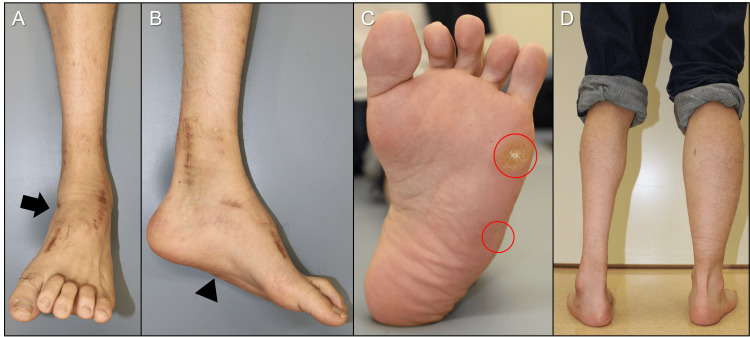
Photographs at 24 months (A) frontal ankle; (B) lateral foot; (C) plantar view; (D) posterior view of both legs in stance. Supination of the foot and ankle has improved (arrow). The medial longitudinal arch has decreased (arrowhead). Plantar callosities have reduced (circles).

During the postoperative course, symptoms improved, and radiographs confirmed the bony union by 3 months. At 24 months, the VAS improved by 100% (7 to 0), the JSSF hindfoot scale improved by 63.5% (55 to 90), and the SAFE-Q scores improved by 171% (33.9 to 91.7) for pain, 73.8% (52.3 to 90.9) for physical function, 300% (25.0 to 100.0) for social function, 83.4% (50.0 to 91.7) for shoe-related, and 200% (30.0 to 90.0) for general health; all categories showed improvement. Cavovarus alignment improved clinically, and plantar callosities reduced (Figures 5A-5D).

**Figure 6:**
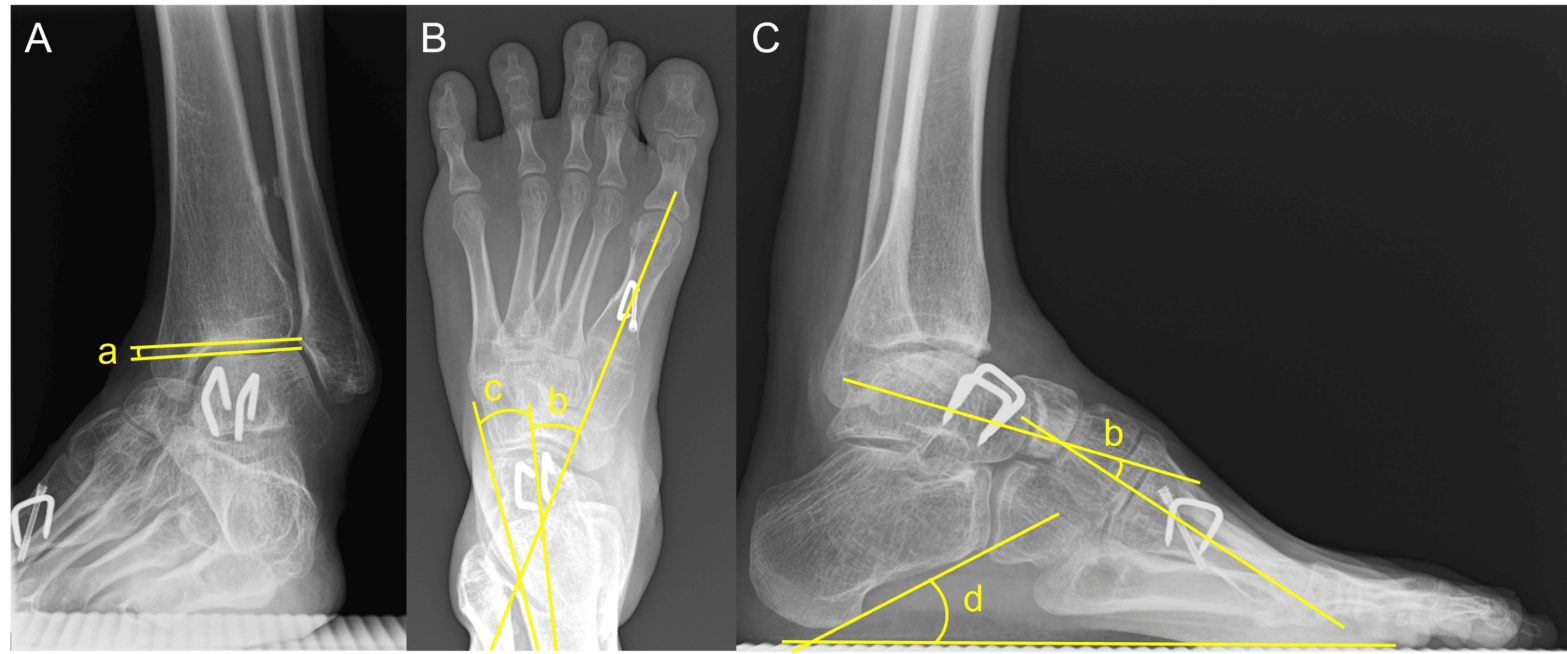
Weight-bearing radiographs at 24 months (A) Anteroposterior view of the ankle; (B) anteroposterior view of the foot; (C) lateral view of the foot. (a) Talar tilt angle; (b) talo-first metatarsal angle; (c) talo-calcaneal angle; (d) calcaneal pitch angle

The ROM of the left ankle joint improved to 25° in dorsiflexion and 45° in plantarflexion. On weight-bearing radiographs, talar tilt angle improved 0.7° (AP ankle), talo-calcaneal angle to 11.2°, talo-first metatarsal angle improved to -28.5° (AP foot), with lateral talo-first metatarsal angle 12° and calcaneal pitch angle 24.5°. Bone union was achieved at the talar and first metatarsal osteotomy site. There were no findings indicative of avascular necrosis (AVN) of the talus, such as sclerosis, deformity, or articular collapse of the talus (Figures 6A-6C).

Table 1 summarizes the pre- and postoperative clinical and radiographic outcomes.

**Table 1:** Pre- and postoperative clinical and radiographic outcomes JSSF: Japanese Society for Surgery of the Foot; SAFE-Q: Self-Administered Foot Evaluation Questionnaire

Parameter	Preoperative	Postoperative (24 months)
Visual analog scale (point)	7	0
JSSF hindfoot scale (point)	55	90
SAFE-Q – Pain (point)	33.9	91.7
SAFE-Q – Physical function (point)	52.3	90.9
SAFE-Q – Social function (point)	25.0	100.0
SAFE-Q – Shoe-related (point)	50.0	91.7
SAFE-Q – General health (point)	30.0	90.0
Talar tilt angle (°)	20.7°	0.7°
AP talo-calcaneal angle (°)	7.2°	11.2°
AP talo–first metatarsal angle (°)	-31.7°	-28.5°
Lateral talo–first metatarsal angle (°)	37.1°	12.0°
Calcaneal pitch angle (°)	9.6°	24.5°

## Discussion

Cavovarus foot has long been associated with neurological diseases such as cerebral palsy, Charcot-Marie-Tooth disease, poliomyelitis, spina bifida, or other hereditary sensory and motor neuropathies [1,2]. Almost all patients with spina bifida experience foot deformities due to congenital, developmental, or iatrogenic causes, with equinocavovarus deformity being the most common [17]. Although deformity correction using external fixators, such as the Ilizarov method, is reported to be less invasive and effective, recurrence is more common in children with spina bifida than in adults, necessitating additional surgery [18,19]. Our patient had previously undergone Ilizarov surgery in childhood; however, the deformity recurred, leading to difficulty in ambulation. Therefore, we determined that surgical intervention was indicated.

Because malorientation of the talar head-neck segment can lead to forefoot adduction and midfoot supination, attention to talar alignment is critical [20]. To correct talar neck malposition, TNO has been performed in pediatric cavovarus feet [8,9] as well as in varus malunion deformities following talar neck fractures [10-12]. However, a report on long-term outcomes of TNO for residual deformities of congenital clubfoot demonstrated poor clinical results, concluding that TNO in children under 10 years of age may cause AVN of the talus and therefore should be abandoned [9]. In contrast, Garcia-Fernandez et al. reported significant improvements in alignment and function, with 100% patient satisfaction, in the mid-term study of TNO combined with tendon transfers and muscle rebalancing for severe neurologic equinovarus feet in nine pediatric patients [8]. They observed only one case of partial, asymptomatic AVN by performing the procedure with meticulous attention to the vascular supply of the talus. In trauma cases, reports on TNO for adolescent or adult cavovarus deformities due to malunited talar neck fractures have demonstrated favorable radiological and clinical outcomes, with few postoperative AVN cases [11,12]. Suter et al. reported that in seven patients, the VAS for pain improved from 7.4 to 1.7 points, and the American Orthopaedic Foot and Ankle Society hindfoot score increased from an average of 40.9 points preoperatively to 83.9 points postoperatively [12]. Similarly, in the present case, VAS improved from 7 to 0, and the JSSF hindfoot score improved from 55 to 90 points. Furthermore, substantial improvements were observed across all domains of the SAFE-Q, indicating not only marked gains in pain and physical function but also notable enhancements in social function and general health. These findings suggest that TNO, when combined with adjunctive joint-preserving procedures, may provide comprehensive benefits that extend beyond structural correction to include broader aspects of quality of life in adults with neurologic cavovarus foot.

Adults with residual or recurrent clubfoot have been reported to exhibit about 9° of increased varus angulation of the talar neck compared with normal feet [20]. A similar trend of talus deformity may also occur in adults with paralytic cavovarus foot. In the present case, the talar neck declination angle relative to the long axis of the talar body was 32.9°on 3D-CT, whereas the average values have been reported as 24° in the cadaveric study [14] and 18.7° on the weight-bearing CT [20]. TNO is less invasive than arthrodesis and allows correction of varus components of the deformity while preserving mobility and function of the talonavicular joint. Therefore, TNO may be an appropriate indication in an adult flexible cavovarus foot with varus deformity of the talar neck, such as in the present case. Although no consensus exists regarding the osteotomy technique (opening wedge vs. closing wedge), corrective angle, or the fixation method, we believe that closing wedge osteotomy using staples is advantageous because it minimizes stress on the soft tissue, avoids complex techniques, and does not require autogenous bone grafting. Major concerns regarding TNO include intraoperative complications, nonunion, development or progression of AVN, and degenerative changes around the talus [10]. To mitigate these risks, it is essential to preserve the vascular supply of the talus intraoperatively and to ensure careful radiographic and clinical follow-up postoperatively.

TPT, DFMO, and ATL are commonly performed procedures for cavovarus foot deformities. Each technique is rarely used in isolation but is often combined with other procedures, and good clinical outcomes have been reported [3-6]. TPT not only reduces the major deforming forces of the cavovarus foot but also compensates for the weakened function of the tibialis anterior and peroneus brevis muscles, thereby improving dorsiflexion and valgus forces [4]. In adult cavovarus feet, the transferred PTT does not cause subsequent planovalgus deformity, as the bony structures and ligaments are usually sufficient to maintain the medial arch and prevent collapse [2]. DFMO is an effective procedure for reducing medial forefoot plantarflexion deformity and lowering the longitudinal arch [3,5]. ATL is also effective for correcting the equinovarus deformity associated with contracture of the Achilles tendon [4].

The present patient was 29 years old, highly active, and demonstrated a flexible foot deformity. For such cases, we believe that joint-preserving surgical approaches should be prioritized. Accordingly, we performed a combination of procedures: TNO for correction of midfoot adduction and plantarflexion, TPT for hindfoot varus, DFMO for plantarflexion of the first ray, and ATL for limited ankle dorsiflexion ROM. Favorable outcomes, including pain relief, functional improvement, and correction of deformity without complications, were achieved at 2 years postoperatively. Continued follow-up is necessary, with careful attention to potential recurrence of deformity and postoperative complications.

## Conclusions

We report a case of TNO combined with adjunctive joint-preserving procedures in an adult patient with a flexible cavovarus foot, which achieved favorable short-term clinical outcomes. While these results are encouraging, they represent a single case, and further studies with larger cohorts and longer follow-up are needed to confirm the generalizability of this approach. Compared with standard treatments such as arthrodesis, TNO can offer the advantage of preserving joint motion and being less invasive, although careful patient selection remains essential.

## References

[1] Nogueira MP, Farcetta F, Zuccon A (2015). Cavus foot. Foot Ankle Clin.

[2] Younger AS, Hansen ST Jr (2005). Adult cavovarus foot. J Am Acad Orthop Surg.

[3] Kroon M, Faber FW, van der Linden M (2010). Joint preservation surgery for correction of flexible pes cavovarus in adults. Foot Ankle Int.

[4] Chen ZY, Wu ZY, An YH, Dong LF, He J, Chen R (2019). Soft tissue release combined with joint-sparing osteotomy for treatment of cavovarus foot deformity in older children: Analysis of 21 cases. World J Clin Cases.

[5] Togei K, Shima H, Tsujinaka S, Hirai Y, Yasuda T, Neo M (2022). Joint preserving procedures for painful plantar callosities in patients with flexible cavovarus foot. Foot Ankle Surg.

[6] Moriwaki D, Nakasa T, Ikuta Y, Kawabata S, Adachi N (2025). Lateral displacement calcaneal osteotomy combined with anterolateral transfer of the tibialis posterior tendon for treating flexible cavovarus foot in an adult patient: A case report. Cureus.

[7] Mann DC, Hsu JD (1992). Triple arthrodesis in the treatment of fixed cavovarus deformity in adolescent patients with Charcot-Marie-Tooth disease. Foot Ankle.

[8] Garcia-Fernández J, Galán-Olleros M, Fraga-Collarte M, Ramírez-Barragán A, Martínez-González C, Martínez-Caballero I (2025). Mid-term outcomes of talar neck trapezoidal osteotomy for correction of severe rigid neurologic equinovarus foot. Foot Ankle Surg.

[9] Huber H, Galantay R, Dutoit M (2002). Avascular necrosis after osteotomy of the talar neck to correct residual club-foot deformity in children. A long-term review. J Bone Joint Surg Br.

[10] Barg A, Suter T, Nickisch F, Wegner NJ, Hintermann B (2016). Osteotomies of the talar neck for posttraumatic malalignment. Foot Ankle Clin.

[11] Rammelt S (2012). Secondary correction of talar fractures: Asking for trouble?. Foot Ankle Int.

[12] Suter T, Barg A, Knupp M, Henninger H, Hintermann B (2013). Surgical technique: Talar neck osteotomy to lengthen the medial column after a malunited talar neck fracture. Clin Orthop Relat Res.

[13] Lamm BM, Stasko PA, Gesheff MG, Bhave A (2016). Normal foot and ankle radiographic angles, measurements, and reference points. J Foot Ankle Surg.

[14] Kelikian AS, Sarrafian SK (2023). Sarrafian's anatomy of the foot and ankle: Descriptive, topographic, functional. https://shop.lww.com/Sarrafian-s-Anatomy-of-the-Foot-and-Ankle/p/9781975160630.

[15] Niki H, Aoki H, Inokuchi S (2005). Development and reliability of a standard rating system for outcome measurement of foot and ankle disorders I: Development of standard rating system. J Orthop Sci.

[16] Niki H, Tatsunami S, Haraguchi N (2013). Validity and reliability of a self-administered foot evaluation questionnaire (SAFE-Q). J Orthop Sci.

[17] Westcott MA, Dynes MC, Remer EM, Donaldson JS, Dias LS (1992). Congenital and acquired orthopedic abnormalities in patients with myelomeningocele. Radiographics.

[18] Zang J, Qin S, P V, Shi L, Qin X (2019). The treatment of neurotrophic foot and ankle deformity of spinal bifida: 248 cases in single center. Journal of Neurorestoratology.

[19] Lee DY, Choi IH, Yoo WJ, Lee SJ, Cho TJ (2011). Application of the Ilizarov technique to the correction of neurologic equinocavovarus foot deformity. Clin Orthop Relat Res.

[20] Malhotra K, Colta R, Jani P, Haldar A, Patel S, Welck M, Cullen N (2024). Talar neck rotation angle in adults with clubfoot deformity: Observed values and intra- and inter-observer reliability using weightbearing CT. Foot Ankle Surg.

